# Bioengineering Approaches for Delivering Growth Factors: A Focus on Bone and Cartilage Regeneration

**DOI:** 10.3390/bioengineering9050223

**Published:** 2022-05-20

**Authors:** Sheeba Shakoor, Eleyna Kibble, Jehan J. El-Jawhari

**Affiliations:** Biosciences Department, School of Science and Technology, Nottingham Trent University, Nottingham NG11 8PT, UK; sheeba.shakoor2021@my.ntu.ac.uk (S.S.); eleyna.kibble2018@my.ntu.ac.uk (E.K.)

**Keywords:** growth factors, bone, cartilage, regeneration

## Abstract

Growth factors are bio-factors that target reparatory cells during bone regeneration. These growth factors are needed in complicated conditions of bone and joint damage to enhance tissue repair. The delivery of these growth factors is key to ensuring the effectiveness of regenerative therapy. This review discusses the roles of various growth factors in bone and cartilage regeneration. The methods of delivery of natural or recombinant growth factors are reviewed. Different types of scaffolds, encapsulation, Layer-by-layer assembly, and hydrogels are tools for growth factor delivery. Considering the advantages and limitations of these methods is essential to developing regenerative therapies. Further research can accordingly be planned to have new or combined technologies serving this purpose.

## 1. Introduction

The bone regeneration process is a physiological process that is ideally constituted of three consecutive stages but with some overlapping. Cartilage regeneration shares the first two stages of bone regeneration. During the inflammation phase, a hematoma initially present at the injury site contains platelets and recruited inflammatory cells to secrete pro-inflammatory cytokines. The immune cells, including macrophages and natural killer cells, also mediate the recruitment of mesenchymal stem cells (MSCs) via cytokines for differentiation and regeneration, while neutrophils and osteoclasts clear debris and damaged tissue [1].

The repair phase involves mesenchymal stem cell differentiation into osteoblasts for primary healing. While in more common secondary healing, the formation of soft callus via mesenchymal stem cell differentiation into chondrocytes occurs to be converted into a hard callus. Angiogenesis is also involved within this phase, facilitated by MSCs. Macrophages are involved in various processes of the repair phase, such as angiogenesis and soft to hard callus formation via the release of type 1 collagen, linked to higher levels of macrosialin protein during hard callus formation. Cytokines released by B and T-lymphocytes such as IL-17 and TNF-α and noted growth factors such as platelet-derived and bone morphogenetic protein growth factors, platelet-derived growth factors (PDGFs) and bone morphogenic proteins (BMPs), regulate soft callus mineralization [1].

In the remodeling phase, woven hard callus bone is regulated by the balanced functions of osteoclast and osteoblast. Both macrophages and MSC functions facilitate the osteoblast effect with activation from several growth factors. However, macrophage osteoclast progenitor activity increases osteoclast differentiation, whereas MSCs release osteoprotegerin to antagonize this differentiation. T-lymphocytes also produce IL-17, a key inducer of hard callus conversion. Osteoclast interactions with osteoblasts are also vital in the remodeling phase; M-CSF, RANKL, TNF-α, and osteal macrophages impact this relationship [2].

Therapeutic growth factors are needed to enhance bone or cartilage repair in some conditions where healing is complicated or delayed. Traumatic bone healing could be complicated in up to 15% of the cases [3]. Several other pathological conditions, such as osteonecrosis and arthritis, are accompanied by degenerative bone lesions [4]. In all these conditions, autologous/intrinsic bone progenitor cells might not be fit for healing and need the enhancing role delivered by growth factors. The most known growth factors that enhance bone and cartilage are discussed below and in Table 1.

There are many bioengineering methods to deliver these growth factors to enhance bone and cartilage regeneration. These methods are essentially needed to support repair cells, particularly in problematic healing. These methods include scaffolds of different types, such as metals, ceramic, and polymers. Another method is encapsulation, which can be done physically or using microparticles or nanoparticles. Layer-by-layer assembly and hydrogel technologies are other popular tools to deliver growth factors [5]. The principle, advantages and limitations of these methods will be discussed in detail in this review.

**Table 1:** Growth factors that enhance bone and cartilage regeneration.

Growth Factors	Source	Effector Cells	Function	Pathways
**TGFβ** [6,7,8,9,10]	Platelets	MSCs Fibroblasts? (Collagen)	Osteogenesis Chondrogenesis Collagen Type 2 synthesis Proteoglycan synthesis	MAPK ERK SAPK/JNK
**BMPs** [11,12,13,14,15,16]	Platelets	MSCs Fibroblasts? (Collagen) Endothelial cells	Osteogenesis Chondrogenesis Collagen Type 2 synthesis Proteoglycan synthesis Angiogenesis	MAPK ERK SAPK/JNK
**VEGF** [17,18]	Osteoblasts Pre-osteogenic cells Chondrocytes	Endothelial cells	Neovascularization Osteogenic cells recruitment	RAS-raf-ERK/1/2 PI3K/AKT
**PDGF** [19,20,21,22]	Platelets	MSCs Chondrocytes Inflammatory Cells	Mitosis Chemotaxis Extracellular Matrix Formation Cartilage formation Osteogenesis	ERK1/2 PI3K/AKT
**IGF** [23,24,25,26]	Osteoblasts Chondrocyte (Hepatocytes)	MSCs Myeloid Precursor Cells Osteoclasts	Osteogenesis Chondrogenesis Osteoclast differentiation Osteoblast chemotaxis Osteoclast function Type 1 collagen release	Mtorc2/AKT ERK1/2 PI3K/AKT
**FGF** [27,28,29,30]	MSCs Osteoblasts Chondrocytes (Inflammatory) Endothelial cells Macrophages	MSCs Osteoblasts Endothelial cells	Chondrogenesis Osteoblast proliferation Angiogenesis Inflammation MSC proliferation Bone formation	PLC3-K/AKT Ras/MAPK PLC PKC STAT1/p21

## 2. Growth Factors Helping Bone and Cartilage Regeneration

### 2.1. Transforming Growth Factor-Beta

Growth factors in the transforming growth factor-beta (TGF-β) superfamily are unified by polypeptide structure, functional with a broad range of target cells that facilitate cellular proliferation, differentiation, extracellular matrix production, and embryonic development. The TGF-β family, particularly isoforms 1–3 (TGFβ1–3), are implicated in cartilage and bone regeneration [6].

TGFβ1 is explicitly an inducer of osteoblast differentiation during bone reformation. The downstream signaling of TGFβ1 involves the p38 mitogen-activated protein kinase (MAPK), extracellular signal-regulated kinase (ERK), and stress-activated protein kinase/c-Jun NH(2)-terminal kinase (SAPK/JNK) pathways, all active mediators in mesenchymal stem cell differentiation [7]. When released from platelets after blood clot formation at the site of bone injury, TGFβ1–3 binds to type 2 heteromeric receptors, which phosphorylate type 1 heteromeric receptors. Subsequent phosphorylation of specific Smad proteins acting as transcription regulators can induce collagen type 2 and proteoglycan synthesis or secretions associated with ossification of the soft callus [8,9].

N-cadherin expression is also induced by the activation of TGFβ1–3, promoting mesenchymal progenitor cell differentiation into chondrocytes during extracellular matrix development. Conflicting studies have examined whether all TGFβ isoforms are equally stimulatory in chondrogenesis, so further elucidation may be necessary. TGFβ1–3 are important regulators of the TGFβ subfamily BMPs [10].

### 2.2. Bone Morphogenetic Proteins

BMPs are highly established osteogenic factors in skeletal research, with BMP-2, -4, and -7 showing clinical promise in bone and cartilage regeneration. Both homodimer and heterodimer configurations of the 15 BMP isoforms are active in mesenchymal stem cell differentiation into osteoblasts or chondrocytes and are inducers independent of a stimulus [11]. BMPs are also implicated in embryogenesis and cell homeostasis in other tissues [12].

Platelets secrete selected isoforms in various bone healing phases, such as BMP-2, -6, and -9 that are involved in osteoblast formation from MSCs and others in osteocyte maturation. BMP-2 release is localized to the injury site but accepted as an angiogenic factor in contribution to bone regeneration [13]. Although BMP-3 inhibits the functions of other BMPs, it is the amplest isoform in adult bone. As with all TGF-β superfamily growth factors in chondrogenesis, the BMP/heteromeric receptor complexes cause Smad messenger phosphorylation for altered transcription and increased collagen type 2 or proteoglycan, therefore inducing cartilage and associated extracellular matrix development [14].

Amongst the most studied BMPs, the BMP-7 isoform, when overexpressed in MSCs, has been shown to increase the release of vascular factors as well as induce osteogenesis from MSCs secretions. There does, however, appear to be an optimal dose of delivery to rats at 50 ug for this function, whereas a more potent dosage does not increase efficacy [15]. A BMP-2/7 heterodimer combination may be a more effective engineered facilitator of bone repair as opposed to either BMP-2 or -7 isoforms. The impact of this engineered growth factor on inflammation should be elucidated further before human clinical use [16]. The regulation of the pleiotropic function of BMPs between osteogenesis, chondrogenesis, and angiogenesis needs to be established experimentally to optimize the best dose and combination of these factors.

### 2.3. Vascular Endothelial Growth Factor

Mediators of angiogenesis, such as the dimeric vascular endothelial growth factor (VEGF), aid the success of bone and cartilage regeneration. VEGF is particularly implicated in neovascularization and, remarkably, the recruitment of osteogenic cells that differentiate into osteoblasts in bone formation. Its recruitment function is supported by VEGF release during callus formation and resorption [17].

The hypoxia-inducible factor-1 is activated under oxygen environments of low partial pressure post-tissue damage. Osteoblasts and pre-osteogenic cells release VEGF, binding to VEGF tyrosine kinase receptors 1 and 2 (VEGFR1–2) on endothelial cells. VEGFR1–2 activation induces notch, RAS-Raf-ERK1/2, and Phosphatidylinositol-3-kinase (PI3K)/Protein kinase B (AKT) signaling and endothelial cell growth and migration [18]. The resultant blood vessels formed to stimulate the recruitment of osteoblasts to the injury site and provide localized oxygen, nutrients, and growth factors relevant to bone maintenance. Usually, cartilage lacks blood vessels. However, chondrocytes have the capability to release VEGF during bone repair [17]. Not only do factors in the TGFβ superfamily mediate VEGF release, but the PDGF also exhibits the same role [19].

### 2.4. Platelet-Derived Growth Factor

PDGF is observed to induce chemotaxis and mitosis amongst MSCs, chondrocytes, and inflammatory cells. Specifically, the synthesis of hyaline cartilage and its extracellular matrix is induced via PDGF [20]. A role in osteogenesis is also suggested by studies on the PDGF-BB of the five isoforms. Usually, hours to 3 days post-bone injury, platelets are trapped between a hematoma formed post-injury and release PDGF, binding to its respective PDGF receptor (PDGFR) [19]. G-protein coupled receptor kinase interacting protein-1 expression is also increased with PDGF [21]. PDGFR-AA, -AB, and -BB binding of the complementary PDGF isoform initiates ERK1/2 and PI3K/AKT signaling downstream of the growth factor/receptor complex. Proliferative growth is subsequently stimulated in MSCs and inflammatory cells during the inflammatory and early soft callus phase [22].

### 2.5. Insulin-like Growth Factors

Both insulin-like growth factors 1 and 2 (IGF1–2) polypeptide isoforms are imperative in the later stages of bone repair post-inflammatory phase. IGFs can induce osteoblast and chondrocyte development from MSCs or osteoclasts from myeloid precursors. IGFs, therefore, mediate anabolic and catabolic processes in bone repair. Both isoforms bind to IGF binding proteins (IGFBP1–6) and IGF1 or IGF2 receptors. Intracellular signaling in relevant target cells induces bone matrix synthesis via type 1 collagen release, although IGF-1 is also implicated in osteoblast chemotaxis and function via target cell interaction [23]. Osteoblast differentiation relates to IGF-1 via activation of the rapamycin complex 2 (Mtorc2)/AKT pathway for the hedgehog and Gli-2-regulated transcription [24]. The release of IGF-1 is also apparent during impaired bone matrix resorption via osteoclasts, the catabolic link to IGFs in bone repair [25]. This conflicting effect of IGFs makes it tricky to use for bone regeneration.

IGF1 and 2 mediate chondrocyte and cartilage extracellular maintenance via signaling for matrix synthesis and inhibition of enzymes in extracellular matrix degradation. Signaling for PI3K/AKT and ERK 1/2 pathway activation is implicated in these events. With IGF-1 the more abundant isoform in skeletal tissue, MSC chondrogenic differentiation is observed with IGF-1 signaling [26]. However, the IGF-1 activity in bone and cartilage tissue is augmented when combined with other growth factors, such as TGF-β.

### 2.6. Fibroblast Growth Factor

Twenty-two homologous polypeptides encompass the fibroblast growth factor (FGFs) family, which is associated with the renewal of many tissues. In terms of skeletal rejuvenation, FGFs are released from MSCs, osteoblasts, inflammatory chondrocytes, endothelial, and macrophage cells [27]. Subsequent endothelial and osteoblast proliferation via FGFs cell surface binding also indirectly promotes angiogenesis; hence FGFs are recognized as angiogenic factors. FGF transmembrane receptors (FGFRs) contain an intracellular tyrosine kinase domain that is phosphorylated with tyrosine residues under ligand binding and induce a cascade of target protein phosphorylation to induce phospholipase C3-kinase/AKT, Ras/MAPK, phospholipase C, and protein kinase C signaling. The transcription of the gene involved in cell proliferation is also mediated by FGFs via the signal transducers and activators of the transcription 1 (STAT1)/p21 pathway [22].

FGF-2 is the best studied of this growth factor family in bone and cartilage regeneration and is identified as an inflammatory cytokine followed by FGF-1 in only bone regeneration. In terms of bone tissue, both growth factors induce soft-callus formation after the inflammatory phase, osteoblast function in bone reformation, and MSC proliferation [28].

Cartilage joint development and matrix homeostasis via chondrocyte production is facilitated by FGFs, particularly FGF-2. The priming mechanism for chondrogenic differentiation is induced prematurely by FGF-2. Specific induction of F-actin element structural changes during monolayer cartilage expansion aids chondrogenesis, confirming FGF-2 function [28].

As with the previous growth factors discussed, FGF delivery in combination with other growth factors in skeletal regeneration enhances results; however, the coupling should be carefully selected. BMP-6 has been shown to suppress selected FGF-2 pathways in chondrogenic differentiation [29]. In turn, FGF-2 could inhibit the TGF-β effect in MSCs, prohibiting its crucial function in cartilage regeneration [30].

## 3. Platelet-Rich Plasma

Platelet-rich plasma (PRP) has recently gained popularity in the field of tissue engineering as a natural source of growth factors. PRP contains three to five times higher concentrations of platelets as compared to normal plasma and holds several growth factors such as PDGF, TGF, IGF, FGF, and VEGF. Due to the enrichment of growth factors, PRP may help to speed up the tissue regeneration process [31]. Platelet-derived growth factors act as messengers and regulators in an array of cell–cell and cell–extracellular matrix (ECM) interactions. Furthermore, it has been demonstrated that there is a linear link between platelet concentrations and the concentration of accessible cytokines at the site of injury, speeding up the repair. For the preparation of PRP, platelets are firstly activated and then combined with immunocompatible substances such as sodium citrate and calcium chloride [32]. The platelet-rich method is cost-effective, demonstrates promising results, is easy to administer, shows no immune rejection problems, and most of all, compatible with the patient’s body because platelets are extracted from the patient itself. While the other side of this delivery method is that the patient may develop some initial infections, there can be tissue damage at the administered site, and the patient may also suffer pain (Yan et al. 2020). Kaur et al. investigated the use of PRP along with carbonated hydroxyapatite (CHA) and reported that the fusion of PRP and CHA as a hybrid scaffold resulted in higher histological bone production [33]. Another study indicated that platelet inclusion in calcium phosphate cement was found to be promising for angiogenesis and osteogenesis [34]. Qiu et al. also demonstrated that PRP along with calcium phosphate cement showed a positive result for bone regeneration in minipigs [35]. Testing the combined PRP and bone marrow aspirate concentrate (BMAC) for the treatment of knee cartilage lesion on a collagen scaffold, promising clinical results were seen [36]. Compared to PRP, recombinant growth factors and engineered growth factors are considered a better alternative as can be processed to have a longer half-life, more stability, and high receptor-binding aiming for better functional effectiveness [37].

## 4. Delivery of Growth Factors via Scaffolds

Scaffolds are 3D structures that provide the site and environment for the development of bone tissue. They help in cell adhesion, cell migration, and proliferation and accelerate bone remodeling. The most popular growth factor delivery techniques are three-dimensional matrices and porous scaffolds, which have been widely studied in clinical and experimental settings. A successful bone tissue engineering system must meet some biological and mechanical requirements [38]. The scaffold had to be cytocompatible, non-toxic, and non-immunogenic, according to biological needs. The scaffolds should be osteoinductive and osteoconductive in nature. Some mechanical conditions should also be met in addition to biological requirements. The scaffold structure should consist of pores interconnected to help in the diffusion of cells. The microporous scaffold provides a large surface area for the interaction of cells and scaffold [39]. To avoid the risk of problems such as post-operative implant-related osteopenia or eventual refracture, the mechanical characteristics of the scaffold should be tuned to be compatible with the host, weight-bearing sites/activities, and type of tissues. Scaffolds are often constructed to mimic the mechanical properties of human cancellous bone, which has a compressive strength of 2–12 MPa and an elastic modulus of 0.1–5 GPa. The scaffold should also present the degree of porosity that is requisite for cell proper growth, spatial organization, and flow of nutrients. It is considered that a porous structure having a mean pore size higher than 300 µm enhances angiogenesis which improves bone repair. Different types of materials have been evaluated to meet the requirements for a successful bone tissue scaffold. The osteogenic properties of metals, polymers, and ceramics, as well as their potential to reinforce the production of new, functional bone, have been reviewed elsewhere [40].

### 4.1. Metal-Based Scaffolds

Metal-based scaffolds have long been utilized for a variety of bone regenerative applications, owing to their mechanical strength and biocompatibility in particular applications requiring load-bearing capacity. Whilst there are alloys and metals, only a handful are used in orthopedic and biomedical applications, such as titanium (Ti), Zirconium, Platinum (Pt), and stainless steel. Although on the other hand, metals have a substantially higher Young’s modulus than natural bone, which can cause stress shielding and resorption of adjacent bone tissue. Increased porosity is a popular way to minimize the stiffness of metallic implants while also improving the probability of vascularization within the scaffold [41]. There are still some drawbacks to using metallic scaffolds, such as non-degradability, tiredness, ion leakage, and infection risk. Furthermore, these scaffolds continue to show a lack of integration with host tissue and frequently result in the production of fibrous tissue, posing a risk to the durability of scaffolds. Lin et al. worked on the controlled release of Mg^2+^ by using a combination of PLGA and nano-Mg oxide alginate microsphere. They reported that enhanced osteoblastic activity and bone regeneration are seen in situ [42].

### 4.2. Ceramics

Owing to osteogenesis ability, ceramics are widely used in bone repair. Ceramics are made porous using 3-D technology and are then used in tissue engineering. Frequently used ceramics include tricalcium phosphate and calcium phosphates because of their great compatibility with those of the human tissues [43]. Ceramics have high hardness, great wear resistance, and biocompatibility, making them commonly used in bone replacement procedures. In addition, compared to metals and polymer-based implants, ceramic implants produce very less wear debris. Nano-ceramic composites have a smaller grain size than micro-ceramic, which contributes to achieving greater overall mechanical, tribological, and biological performance within the body. Bio-ceramics for tissue engineering can be categorized into Bioinert (zirconia and alumina) and Bioactive (Bioactive glass, Hydroxyapatite (HA), and glass ceramics) based on how they interact with the human body. The advantages of using Bioinert materials are that they exhibit tissue regeneration, biocompatibility, and enhance mechanical strength, but the downside is that they show steady crack growth, exhibit hydrothermal aging, and are expensive in nature. Bioactive materials provide bone formation and represent great stability but are brittle in nature and are hard to fabricate. Lim et al. used bioactive HA scaffolds and, after eight weeks, found that scaffolds possess great bone regeneration and mechanical strength when they are incorporated with a suitable concentration of growth factors [44]. Liu et al. reported that by using 3-D printed bioactive ceramics along with nano-hydroxyapatite bone augmentation, the formation of capillaries and new bone formation is observed [34]. Another study was done in which rabbits were used as a model, and a porous biphasic calcium phosphate microsphere along with collagen was used. The results showed that the microsphere enhances the regeneration of bone during in vitro studies [45].

### 4.3. Polymers

Polymers are generally regarded as an excellent candidate for the regeneration of bone tissues. They are biocompatible, cheap, and easy to process. Polymers are categorized into two classes, natural and synthetic.

Natural polymers are getting attention nowadays owing to their favorable properties like decreased immunogenicity, and low cytotoxicity, and due to their similarity to the extracellular matrix (ECM). There are different sources, including plants and animals, from where polymers are extracted. The commonly used natural polymers for bone tissue engineering are chitosan, silk fibrin, alginate, and hyaluronic acid. These polymers have high biocompatibility, degradability, and less immunogenicity, and they have proven their ability to aid in cell growth. Collagen is one of the most studied polymers for tissue engineering as it is an essential component of the extracellular matrix and has domains for bone cell adhesion. It promotes bone progenitor cell growth and mineral production, as reported in human in vitro studies [46,47]. The eminent feature of collagen is that it is processed in different physical forms and shows enzymatic biodegradability. The limitation of using collagen is that it exhibits poor mechanical strength and is expensive [38]. Calabrese et al. tested the osteogenic collagen ability using human stem cells isolated from adipose tissue and the results demonstrated increased osteogenesis and bone augmentation in mice [48]. Alginate (anionic in nature) has been used in bone tissue engineering due to its cytocompatibility and regulated gelation. Another commonly used natural polymer approved by FDA is Fibrin. It induces angiogenesis and osteogenic differentiation. It shows excellent mechanical properties and high versatility. Fibrin is an integral aspect of bone regeneration since it is a key element in wound healing. The downside of using fibrin is that it exhibits a high shrinking ability, increased degradation rate, and less mechanical stability. Chitosan is another great candidate for bone regeneration as it promotes cell proliferation and adhesion. It promotes bone mineralization, and osteoconductivity and shows antibacterial properties [49].

Fasolino et al. used chitosan-based scaffolds and reported that the scaffolds could prevent the rate of inflammation and decrease oxidative stress in in-vitro models [50]. Another study also showed that bone healing was seen in rabbits [51]. In a study, fibrin was conjugated with heparin to release BMP-2 in rabbits. The results showed the sufficient formation of bone, and side effects such as ectopic bone formation were also reduced. The obtained results were similar to that of autograft, which is considered a gold standard for the regeneration of bone [52]. A group of researchers have created human-like collagen and used it to release BMP-2 in mouse and rat models. The results indicated that it repairs the defects of bone more rapidly and efficiently [53].

Synthetic polymers are useful in the delivery of growth factors because of their tunable properties. Depending upon their applications, their properties can be modified. Furthermore, these polymers are trustworthy source materials. For bone tissue engineering, poly (lactic acid), poly (glycolic acid), poly (lactic–glycolic acid) (PLGA), and polycaprolactone (PCL) are commonly used. The most commonly used synthetic polymer is PCL, and it is approved by FDA and is cost-effective. Furthermore, it shows good properties such as great mechanical strength, elasticity, non-toxic, and higher biodegradation rate. For cell proliferation and cell adhesion, PCL is usually used. The main limitation of using PCL is its hydrophobicity which hinders cell adhesion and infiltration. Great cellular interactions have been seen when PCL is coated with collagen or alginate [54]. Polylactic acid (PLA) is also proved to be an effective polymer having great thermal stability and mechanical properties. In vivo studies showed that PLA is degraded directly by hydrolysis without the action of an enzyme or catalyst. Another FDA-approved synthetic polymer is PLGA which is also biodegradable and biocompatible, but its applications are limited owing to its poor osteoconductive [49].

Despite its advantages, synthetic polymer interactions with the host tissues are limited due to the lack of bioactivity. This property is more apparent when the comparison with natural polymer is made as they have ECM binding domains helping the regeneration of tissues absent in synthetic polymer. However, there are many solutions to work out this drawback, e.g., surface functionalization, especially plasma coatings. Alba-Perez, et al. developed a coating method for bioactive polymer using poly(ethyl acrylate) (PEA) using a custom-made plasma polymerization reactor. This PEA-coated polymer worked by unfolding fibronectin upon adsorption and helped the GF binding region, such as integrin to sequester and present BMP-2 effectively [55]. Furthermore, other studies reported that attachment of PRP covalently or ionically onto plasma polymers showed enhanced effects of these scaffolds. The coating of PCL nanofibers by PRP was found to significantly promote the viability and proliferation of human mesenchymal stem cells [56]. In a recent study, the deposition of plasma-coated polymer layers onto polycaprolactone nanofibers has been shown to enhance the healing of a diabetic wound [57]. Together, these studies indicate that the plasma coating is a great tool to improve the quality of using polymers for the delivery of growth factors. Another method to improve GF factor delivery could be achieved by using a composite of synthetic polymer with natural polymer [38]. Zou et al. used a hybrid polymer, and the results showed excellent biocompatibility. The in-vitro results showed that great mechanical and biocompatibility is achieved by combining chitosan, collagen, polylactic acid, and nano-hydroxyapatite [58]. PLGA and PLGA were used with gelatin nanofibers scaffolds and reported that these matrices lead to enhance cell proliferation and attachment [59]. The results indicated 2.5%–3% enhanced cell proliferation. In another study, the researchers developed 3-D PLA scaffolds and coated them with mucic acid and gelatin. They used a mouse as a test subject, and the results showed improved osteoblast differentiation along with this enhanced expression of Runx2 (bone transcription factor) was seen [60].

## 5. Encapsulation Technology for Growth Factor Delivery

When growth factors are introduced into the cells, they suffer rapid degradation. To avoid them from degradation, they are encapsulated within different biomaterials. This encapsulation allows target specificity, enhanced retention time, and protection from enzyme degradation. Furthermore, regulated dosage restrains inflammatory effects, cytotoxicity, ectopic bone regeneration, and all the difficulties associated with direct protein inoculation [61]. So, different encapsulation methods are used to avoid these hindrances, as discussed below.

### 5.1. Physical Encapsulation

Physical encapsulation of growth factors is a great technique to prevent protein degradation. Different physical encapsulation techniques have been developed, such as phase emulsion, freeze-drying solvent casting, and gas foaming [62]. The main challenge facing this fabrication process is that the growth factors get exposed to harmful solvents. The gas foaming technique can be employed to avoid this process as it does not contain any solvent. The scaffold’s final structure depends upon the gas and polymer material, with carbon dioxide has been preferred for porosity. The disadvantage of using physical encapsulation is the burst release. Currently, liposomes seem to be a promising choice for the encapsulation of growth factors as they exhibit high tolerance and efficient loading capacity. However, it is reported that this system shows instability in the physiological environment [63].

### 5.2. Microparticles

Due to their properties, microparticles (MPs) act as an exceptional carrier for growth factor delivery. They have high drug loading ability, enhanced diffusion, and a high surface-to-volume ratio. MPs have been traditionally used as drug delivery carriers. When the MPs containing growth factors encounter water, MPs get swell as the water molecule enters the insides of the MPs, allowing the growth factor to encounter the outside environment. In the meantime, the MPs are degraded, and the growth factors are released [64]. The rate at which the growth factors are released can be controlled. When MPs loaded with growth factors are immobilized on the surface of scaffolds, they can govern the release of growth factors without changing the scaffold structure. Various polymers such as collagen, alginate, silk, and gelatine show biocompatibility and can be used to deliver MPs. Gelatine, a natural polymer, is successfully being used to incorporate TGF-β1. Acid gelatine represents a higher affinity for positively charged growth factors. The limitation of using natural polymers is that it is difficult to control the degradation rate, but several additional modifications are required [65]. Synthetic polymers MPs are also in use for decades for the delivery of growth factors. PLGA is the most popularly used and efficient synthetic polymer for growth factor delivery. While synthetic polymer-based MPs offer the edge of attaining defined release kinetics, they also carry the risk of causing an inflammatory tissue response and altering the bioactivity of growth factors. A study showed that hyaluronic acid-based particles are synthesized, which contain immobilized heparin. The particles provide the controlled release of BMP-2 [66].

### 5.3. Nanoparticles

Nanoparticles (NPs) are another delivery system for growth factors that has been gaining a lot of popularity. The phase control of monometallic and alloy nanomaterials has been received recently, demonstrating the ongoing advanced research to improve their synthesis methods and, consequently applications [67]. The releasing mechanism of NPs is similar to that of MPs, but they offer versatility as they have the ability to release the encapsulated growth factors inside or outside of the target cells. They have a great surface area to volume ratio so they can hold more growth factors inside them [68]. There are numerous advantages of using NPs as a growth factor carrier, including overcoming mechanical property restrictions. Incorporating nanoparticles into scaffolds has been shown to enhance the scaffolds’ mechanical strength. Studies confirmed that nanoparticles provide a sequential and sustained release of growth factors for bone regeneration. A study was conducted in which BMP-7 loaded NPs were used in rabbits, and the results showed that controlled release of BMP-7 subsequently enhances the collagen and results in the formation of thick hyaline cartilage [69]. Although there are numerous advantages of NPs in drug delivery, their intrinsic properties also cause some obstacles. A high surface area to volume ratio enhances the growth factor loading capacity in NPs, but it may also result in the reduction of NP stability.

A recent study described how high-density lipoprotein loaded with a chemotherapeutic drug can act as a therapeutic delivery tool. The encapsulated particles can be specifically delivered into the cells and show a successful anti-cancer effect. The system incorporates the ability to impart photodynamic therapy where laser light would not be accessible [70].

## 6. Layer by Layer Assembly Technology for Growth Factor Delivery

Layer by layer is a simple and easy technique for the formation of polyelectrolyte multilayer. Porous scaffolds are generally modified through this process for the controlled delivery of growth factors because it performs well in preventing growth factor function loss and sequestering high growth factor concentration in a moderate aqueous environment. In the formation of this assembly, hydrogen bonding, electrostatic and covalent interactions are typically utilized [71]. The polyelectrolyte multilayer properties can be adjusted to regulate the growth factor release. Therefore, this assembly technique enhances loading capacity and desired bio-factor release can be used for the delivery to the target site. Vectors for gene delivery and DNA can be introduced into the layers without any alteration to their native conformation. 3-D bioprinting is a novel LBL technique that combines materials with growth factors and forms a 3-D scaffold, thus, manifesting capability in the framework of regulated drug delivery [72].

Ansboro et al. worked on the LBL technique and reported that TGF-B-3 binds the HA microsphere, presents an appropriate drug delivery system, and enhances the chondrogenic gene expression [73]. Another study exhibits that on the PLGA membrane, an LBL nanolayer coating of BMP-2 and PDGF resulted in better bone regeneration than BMP-2 alone delivery in mice models [74].

## 7. Hydrogel Technology for Growth Factor Delivery

Hydrogels are compounds with a high-water content and are among the few biomaterials that can be utilized to make ECM-like scaffolds. Hydrogels can be utilized for controlled drug delivery to target sites in bone defects [75]. Drug encapsulation in the hydrogel is one of the common and simple strategies to produce a 3-D drug delivery system. Hosting of drugs, proteins, and DNA within hydrogel can be done by mixing the polymer matrix before any kind of crosslinking. For hydrogel carriers, both natural and synthetic materials can be used [76]. Hydrogel releases drugs on the desired sites and possesses tissue-compatible substrates for better cell growth and attachment. Hydrogels could preserve the growth factor bioactivity for a long duration preventing them from environmental degradation. It has been reported that BMP-2 incorporated inside HA hydrogel remained bioactive even after 28 days. Another study demonstrated that BMP-2 had been encapsulated in the gelatin implants, and they remained active for six weeks in the cell culture [77]. In another study, a hydrogel coating on the surface of the PLC scaffold was utilized for BMP-2 delivery for checking its effect on bone regeneration. The results showed enhanced bone mineralization and regeneration in contrast with scaffolds without containing hydrogel [78]. Hydrogel also has some limitations. A molecule’s entrapment directly depends upon which encapsulation method is used. In the physical encapsulation method, usually, diffusion is used for the controlled release, while in chemical encapsulation, the release depends on polymer degradation or gel matrix dissociation. Another disadvantage is that the hydrogel network mainly consists of water, so its tensile strength may be lower, hindering its capacity to bear the load [79].

## 8. Conclusions

Growth factors are a promising tool for enhancing bone and cartilage tissue repair. Growth factors bind to transmembrane receptors, specifically to the extracellular region on a target cell associated with bone and cartilage tissue. The intracellular cascade induced and subsequent responses in cell behavior are determined by various characteristics of each growth factor, enforcing specificity to each growth factor delivery system that can be engineered for clinical application. Certain tissue engineering strategies are used to deliver human growth factors to the damaged cartilage or bone tissues. Direct distribution of growth factors and cells has demonstrated considerable therapeutic value in preclinical and clinical trials, but only a few are available as products for clinical applications.

Growth factors stimulate several biological activities during growth and tissue repair, including cell proliferation, migration, and differentiation; as a result, they have sparked a lot of interest in regenerative medicine applications, and various growth factor-based products have been produced. Recombinant growth factors are associated with the targeted and slow or timely release of the protein at the site of injury. These factors not only play a major role in regulating the time and dose-dependent release of the protein but also help in cell proliferation and support cell migration at the target site [80]. Different growth factors such as bone morphogenetic protein BMP-7 and BMP-2 are used for bone regeneration and showed good results. BMP-7 is approved by the FDA for clinical usage [81]. The extremely short intervals of biological activity and limited time span of effectual local concentrations are common limits of this method. Another limitation is that, due to the presence of proteolytic enzymes, the administered protein may alter its structure [80]. In a recent study, Zhou et al. investigated the outcome of recombinant six human FGF-18 (rhFGF-18), and the results showed that in rat models, it improved the tendon to bone healing by stimulating fibrocartilage regeneration [82].

**Figure 1:**
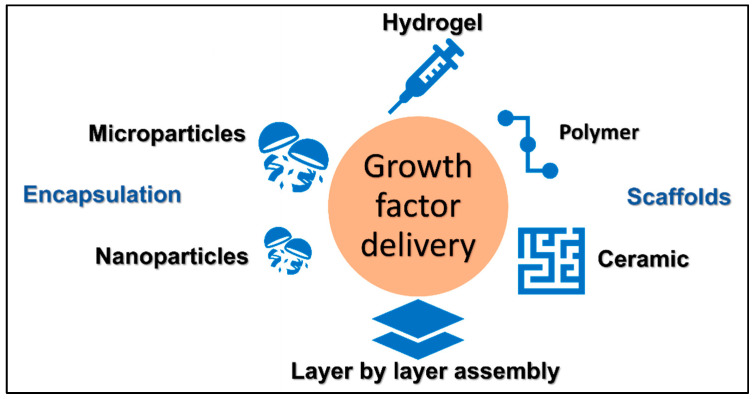
Methods for the delivery of growth factors.

Despite having great potential in bone regeneration, the clinical use of growth factors is still limited because of their short time span, side effects, and high cost. Different methods for delivery are in use for research (Figure 1) with each having advantages and disadvantages (Table 2). Using scaffolds of different types is still a practical method for growth factor delivery, but degradation by enzymatic or biological mechanisms within the host is a strong barrier reducing the effectiveness. Hosting and protecting the growth factors using encapsulation, the LBL method seems to be a better option for prolonged growth factor half-life. Some safety issues have been reported as a result of using growth factors such as inflammation, overgrowth of bone, cancer, nerve damage, and immune response [83]. Localized and customized delivery is needed for ensuring safety when applying these growth factors as regenerative aids. Along with the side effects, the cost is also another hurdle in using these growth factors. Using growth factors instead of conventional ways costs more [84]. Therefore, more studies are required to make the process more cost-effective. Future research could be directed to combine technologies to host growth factors and support reparatory cells simultaneously.

## Figures and Tables

**Table 2:** Bioengineering methods of growth factor delivery, examples, advantages, and disadvantages.

Method	Examples	Advantages	Disadvantages	Ref.
**Scaffolds**	Polymers: synthetic, natural, mixed, plasma coated	Mechanical stability Easy to fabricate	Natural polymers: low mechanical stability.	[46,47,48,49,50,51,52,53,54,55,56,57,58,59,60,61,62,63,64,65,66,67,68,69,70]
	Ceramic Bioinert: zirconia and alumina, Bioactive: Bioactive glass, Hydroxyapatite, glass	Biodegradable Biocompatible	Bioinert ceramics exhibit Hydrothermal aging Brittle	[43,44,45]
	Metal-based scaffolds Titanium, Zirconium, Platinum, stainless steel	High Young’s modulus Enhanced compressive strength	Ion release Non-degradable	[41,42]
**Encapsulation**	Physical encapsulation: Phase emulsion, freeze-drying solvent casting, gas foaming	Maintained bioactivity	Rapid burst release	[62,63]
	Microparticles Synthetic polymers MPs: PLGA	High surface to volume ratio High drug loading ability	Difficult to control the degradation rate	[64,65,66]
	Nanoparticles: BMP-7 loaded NPs	Controlled release Enhance mechanical strength	Unstable in nature	[67,68,69,70]
**Layer-by-layer** **Assembly**	3-D bioprinting	Retains growth factor functions High tensile strength	Low loading capacity Bone overgrowth	[71,72,73,74]
**Hydrogel**	HA hydrogel, Collagen, Chitosan, Alginate	Site-specific release	Low tensile strength	[75,76,77,78,79]

## Data Availability

Not applicable.
